# Gut microbiota signatures in *Schistosoma japonicum* infection-induced liver cirrhosis patients: a case–control study

**DOI:** 10.1186/s40249-021-00821-8

**Published:** 2021-03-26

**Authors:** Qi-Feng Gui, Hui-Lin Jin, Feng Zhu, Hai-Feng Lu, Qin Zhang, Jia Xu, Yun-Mei Yang, Chi Xiao

**Affiliations:** 1 Department of Geriatrics, The First Affiliated Hospital, School of Medicine, Zhejiang University, Hangzhou, Zhejiang People’s Republic of China; 2 Zhejiang Provincial Key Laboratory for Diagnosis and Treatment of Aging and Physic-Chemical Injury Diseases, The First Affiliated Hospital, School of Medicine, Zhejiang University, Hangzhou, Zhejiang People’s Republic of China; 3 Department of Geriatrics, Wangdian People’s Hospital, Jiaxing, Zhejiang People’s Republic of China; 4 State Key Laboratory for Diagnosis and Treatment of Infectious Diseases, Collaborative Innovation Center for Diagnosis and Treatment of Infectious Diseases, The First Affiliated Hospital, College of Medicine, Zhejiang University, Hangzhou, People’s Republic of China; 5 Department of Emergency Medicine, The First Affiliated Hospital, School of Medicine, Zhejiang University, Hangzhou, Zhejiang People’s Republic of China; 6 School of Basic Medical Sciences & Forensic Medicine, Hangzhou Medical College, Hangzhou, Zhejiang People’s Republic of China

**Keywords:** Gut microbiota, 16S rRNA, *Schistosoma japonicum*

## Abstract

**Background:**

Several studies have assessed the role of gut microbiota in various cirrhosis etiologies, however, none has done so in the context of *Schistosoma japonicum* infection in humans. We, therefore, sought to determine whether gut microbiota is associated with *S. japonicum* infection-induced liver cirrhosis.

**Methods:**

From December 2017 to November 2019, 24 patients with *S. japonicum* infection-induced liver cirrhosis, as well as 25 age- and sex-matched controls from the Zhejiang Province, China, were enrolled. Fecal samples were collected and used for 16S rRNA gene sequencing (particularly, the hypervariable V4 region) using the Illumina MiSeq system. Wilcoxon Rank-Sum and PERMANOVA tests were used for analysis.

**Results:**

Eight hundred and seven operational taxonomic units (OTUs) were detected, of which, 491 were common between the two groups, whereas 123 and 193 were unique to the control and cirrhosis groups, respectively. Observed species, Chao, ACE, Shannon, Simpson, and Good’s coverage indexes, used for alpha diversity analysis, showed values of 173.4 ± 63.8, 197.7 ± 73.0, 196.3 ± 68.9, 2.96 ± 0.57, 0.13 ± 0.09, and 1.00 ± 0.00, respectively, in the control group and 154.0 ± 68.1, 178.6 ± 75.1, 179.9 ± 72.4, 2.68 ± 0.76, 0.19 ± 0.18, and 1.00 ± 0.00, respectively, in the cirrhosis group, with no significant differences observed between the groups. Beta diversity was evaluated by weighted UniFrac distances, with values of 0.40 ± 0.13 and 0.40 ± 0.11 in the control and cirrhosis groups, respectively (*P* > 0.05). PCA data also confirmed this similarity (*P* > 0.05). Meanwhile, the relative abundance of species belonging to the Bacilli class was higher in cirrhosis patients [median: 2.74%, interquartile range (IQR): 0.18–7.81%] than healthy individuals (median: 0.15%, IQR: 0.47–0.73%; *P* < 0.01), and that of Lactobacillales order was also higher in cirrhosis patients (median: 2.73%, IQR: 0.16–7.80%) than in healthy individuals (median: 0.12%, IQR: 0.03–0.70%; *P* < 0.05).

**Conclusions:**

Cumulatively, our results suggest that the gut microbiota of *S. japonicum* infection-induced liver cirrhosis patients is similar to that of healthy individuals, indicating that bacterial taxa cannot be used as non-invasive biomarkers for *S. japonicum* infection-induced liver cirrhosis.

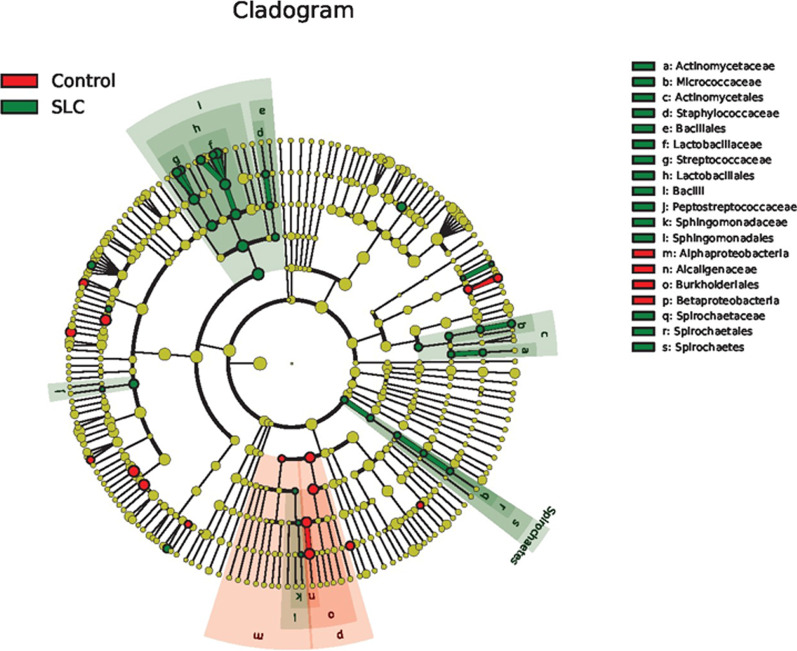

## Background

Schistosomiasis is one of the most prevalent parasitic diseases in the world [1]. According to the World Health Organization (2020), an estimated 240 million people are affected by this disease, together with an additional 700 million at risk of infection [2]. Worryingly, schistosomiasis alone is associated with a high disease burden, of as much as 3.3 million disability-adjusted life years, accounting for more than 10% of the global burden of neglected tropical diseases [3, 4]. Five main etiologic agents have been described for schistosomiasis, all of which are trematode parasites of *Schistosoma* genus, including *S. japonicum*, *S. mansoni*, *S. haematobium*, *S. intercalatum*, and *S. mekongi* [5]. However, only *S. japonicum* exists in China, and is predominant in 12 provinces south of the Yangtze River [6]. In the 1950s, an estimated 11.6 million people were infected, with more than 100 million at risk of infection [7]. From the mid-1950s, the number of infected cases was reduced by over 99%; however, schistosomiasis remains endemic in 140 counties in China [1].

One of the serious complications of *S. japonicum* infection is the development of liver cirrhosis [8]. Most egg-laying adult *Schistosoma* trematodes have liver tropism, and the local (hepatic) host immune responses to the deposited eggs result in granuloma formation, which ultimately evolves to progressive liver fibrosis and portal hypertension [9]. Both in vivo and clinical studies focusing on *S. japonicum* infections revealed that hepatic stellate cells are the key drivers of hepatic fibrosis [8, 10], via collagen production and deposition. Importantly, liver fibrosis presentations have the potential to evolve into liver cirrhosis or liver cancer [11]. Several studies have assessed the role of the gut microbiota in cirrhosis of different etiologies [12–16] with microbiota dysbiosis having been reported in cirrhosis patients as follows: (i) autochthonous/non-autochthonous taxa ratio reduction, (ii) Firmicutes/Bacteroidetes ratio inversion, or (iii) increased prevalence of potentially pathogenic bacteria [17–19]. Interestingly, the liver-gut axis is involved in the physiopathology of cirrhosis; specifically, overgrown intestinal bacteria have the potential to translocate across the leaky gut barrier and reach the liver, leading to cirrhosis development or progression [20].

Recent studies using not only animal models, but also human clinical samples have demonstrated a clear relationship between gut microbiota alterations and *Schistosoma* spp. infection [21–26]. However, no study has explored the relationship between the gut microbiota and *S. japonicum* infection-induced liver cirrhosis. Accordingly, the potential role of gut microbiota alterations in disease onset and progression remains unclear.

Here, we hypothesized that an association between gut microbiota and *S. japonicum* infection-induced liver cirrhosis exists. To explore this hypothesis, a case-control study was conducted. The main objectives of this study were as follows: (1) to characterize the potential gut microbiota alterations associated with *S. japonicum* infection-induced liver cirrhosis; (2) to identify whether there are potential bacterial taxa that can be used as non-invasive biomarkers of liver cirrhosis secondary to schistosomiasis.

## Methods

### Ethics statement

This study was conducted according to the principles expressed in the Declaration of Helsinki. All experiments were approved by the First Affiliated Hospital Clinical Research Ethics Committee, School of Medicine, Zhejiang University (ref: 2017411-1), and by the Jiaxing Wangdian People's Hospital Ethics Committee (ref: 2017002). All participants provided written informed consent.

### Subject enrollment

The *S. japonicum* infection-induced liver cirrhosis patient inclusion criteria were as follows: (i) diagnosis of schistosomiasis performed using the Kato-Katz method to detect *S. japonicum* eggs in stool samples; (ii) diagnosis of liver cirrhosis according to clinical and biochemical data, as well as imaging examination [computed tomography (CT) or B-ultrasound] [27, 28]; (iii) age ≥ 18 years. Eligibility was defined by the application of the following exclusion criteria: (i) history of digestive disorders, including other causes of liver disease*,* irritable bowel syndrome, inflammatory bowel disease, or chronic diarrhea; (ii) history of previous gastrointestinal surgery; (iii) history of probiotic, prebiotic, synbiotic, antibiotic, acid suppressor, metformin, or gastrointestinal motility targeting-drug use less than eight weeks before enrollment; (iv) history of bowel preparation, less than four weeks before enrollment; (v) diagnosis of major diseases that can affect the gut microbiota, such as malignant tumors, cardiovascular, respiratory, autoimmune, or allergic diseases, neurological disorders, renal insufficiency, diabetes, uncontrolled hypertension (blood pressure ≥ 150/90 mmHg), dyslipidemia, depression, mania, and bipolar affective disorder; (vi) hepatitis B virus, hepatitis C virus, or HIV infection.

Due to the lack of data on effect size, power analysis could not be performed. However, a recent similar study on *S. japonicum* infection in which each group consisted of 24 animals supports the appropriateness of our sample size [24]. From December 2017 to November 2019, 24 patients with *S. japonicum* infection-induced liver cirrhosis were enrolled from Hangzhou and Jiaxing, northern cities of the Zhejiang Province, China, a schistosomiasis japonicum endemic area. Liver cirrhosis patients (either inpatients or outpatient) conforming to all inclusion criteria and not meeting any of the exclusion criteria were enrolled by specified physicians. All 24 patients included in this study were previously infected (neither currently infected nor recurrently infected). Eleven patients were treated with praziquantel in the acute infection period, whereas eight were not treated due to patient refusal of treatment for no or mild symptoms; for the remaining five patients, no information was available with respect to schistosomiasis treatment. In addition, 26 age- and sex-matched healthy individuals not meeting any of the exclusion criteria were enrolled from Hangzhou and Jiaxing. Since the stool specimen of one healthy individual was deemed unsatisfactory (stool mixed with blood), the healthy control group was ultimately composed of 25 individuals. The demographic information, laboratory tests, CT, and B-ultrasound results from all participants were collected from the hospital electronic medical records system. Additionally, questionnaires were filled by all subjects to obtain their sex, age, marriage status, occupation, education level, diet, eating behavior, exercise habits, and smoking or alcohol use status. Questionnaire data quality was controlled through special training of investigators on objectives, filling, and administration methods. Liver function was evaluated using the Child–Pugh classification system [29] and was based on five parameters, including ascites, hepatic encephalopathy, serum albumin, total bilirubin (TB), and prothrombin time (PT). Child–Pugh scores were calculated and defined as follows: grade A, 5–6 points; grade B, 7–9 points; grade C, ≥ 10 points.

### Fecal sample collection

The participants were trained by a researcher to obtain a complete stool sample. Fresh stool samples (self-collected by patients and controls) were collected into anaerobic bags (Mitsubishi Gas Chemical, Tokyo, Japan). Samples were divided into five 200-mg aliquots, within the first 30 min after collection, and immediately stored at − 80 °C until further analysis.

### Genomic DNA extraction

Total genomic DNA was extracted per sample using the MagPure Stool DNA KF kit B (Magen, Guangzhou, China) according to the manufacturer’s instructions. DNA was quantified using the Qubit Fluorometer (Qubit 2.0, Invitrogen, CA, USA) and the Qubit® dsDNA BR Assay kit (Invitrogen). DNA quality was assessed using gel electrophoresis (1% agarose gels). Negative controls containing elution buffer only were included for DNA extraction and quantification to monitor possible contamination.

### Library construction and sequencing

The bacterial 16S rRNA gene hypervariable region V4 was amplified using degenerate polymerase chain reaction (PCR) with the primers 515Fw (5′-GTGCCAGCMGCCGCGGTAA-3′) and 806Rv (5′-GGACTACHVGGGTWTCTAAT-3′), which contained the Illumina adapter sequence, a pad, and a linker. Fifty-microliter reaction volumes, containing 30 ng of DNA templates together with the primers (10 μmol/L) and the PCR master mix, were used. Amplification was performed under the following conditions: 95 °C for 3 min; 30 cycles of 95 °C for 45 s, 56 °C for 45 s, and 72 °C for 45 s; a final extension step at 72 °C for 10 min. PCR products were purified using Agencourt AMPure XP beads (Beckman Coulter, CA, USA) according to the manufacturer’s instructions. Library quality was evaluated using the 2100 Bioanalyzer (HT DNA 1K Labchip 760517) instrument (Agilent Technologies, Santa Clara, CA, USA). Sequencing was performed using the Illumina HiSeq 2500 platform (BGI, Shenzhen, China) following the Illumina standard pipelines to obtain 2 × 250 bp paired-end reads.

### Bioinformatics analysis

Raw reads were filtered to remove adaptors and low-quality and/or ambiguous bases, and paired-end reads were merged using the Fast Length Adjustment of Short reads (FLASH, v1.2.11) software [30]. Sequences were clustered into operational taxonomic units (OTUs) with a cutoff value of 97% using the UPARSE software (v7.0.1090) [31]. Chimeric sequences were detected (compared with the Gold database) using UCHIME software (v4.2.40) [32] and removed from the analysis. Sequences of representative OTUs were then taxonomically classified using the Ribosomal Database Project Classifier (v.2.2) with a minimum confidence threshold of 0.6 and trained on the Greengenes database (v201305) using the QIIME pipeline (v1.8.0) [33]. The USEARCH_global command [34] was used to quantify the abundance of OTUs in each sample.

### Bacterial diversity analysis

Alpha and beta diversities were estimated using MOTHUR software (v1.31.2) [35] and the QIIME pipeline (v1.8.0) [33], respectively. Sample clustering was conducted using the QIIME pipeline (v1.8.0) [33] and the unweighted pairwise grouping method with averaging. Barplots and heatmaps of different classification levels were plotted with the R package (v3.4.1) “heatmap” and “gplots” tools, respectively. Venn diagrams and accumulation curves were obtained using the R package “VennDiagram” (v 3.1.1) tool. A principal component analysis (PCA) was performed using the R package “ade4” tool. Taxonomic characterization was performed using a linear discriminant analysis (LDA) with the calculation of the LDA effect size (LEfSe).

### Statistical analysis

Continuous variables are presented as the mean ± standard deviation (SD) or median [interquartile range (IQR)], while categorical variables are presented as percentages (%). The Student’s *t*-test or Wilcoxon Rank-Sum test and the *χ*^2^ test were used to evaluate differences between two groups for continuous and categorical variables, respectively. Whenever applicable, the false discovery rate (FDR) was used to correct the calculated *P*-value. Statistical analyses were performed using SPSS software (v 22.0; SPSS, IL, USA). Statistical significance was given by *P* < 0.05, FDR < 0.05, or LDA scores > 2.

## Results

### Participant characterization

The study population included a total of 24 patients with *S. japonicum* infection-induced liver cirrhosis (12 males and 12 females), and 25 age- and sex-matched healthy individuals (12 males and 13 females). The average ages of cirrhosis and control groups were comparable, 82.7 ± 7.3 and 82.3 ± 7.1 years, respectively (Table 1); in fact, since this was a case-control study with matched controls, no differences in terms of age or sex were expected. Furthermore, no significant differences were observed concerning body mass index, or smoking and drinking habits between the two groups. In addition, the platelet counts, and serum levels of TB, direct bilirubin, and alanine aminotransferase were comparable between the groups. However, whereas the serum levels of aspartate aminotransferase (AST) and glutamyl transpeptidase (GGT), together with the PT were significantly increased in the *S. japonicum* infection-induced liver cirrhosis group, the total protein, and albumin serum levels were significantly decreased in the same group, in comparison with those in the control group (Table 1; at least *P* < 0.05). Regarding disease severity stratification, performed only for the cirrhosis group, 13 patients (54.2%) were classified as Child–Pugh A, seven (29.2%) as Child–Pugh B, and four (16.7%) as Child–Pugh C (Table 1).

**Table 1:** Demographic and clinical characteristics of the study population per group

Variables	SLC group	Control group	*P* value
*n*	24	25	
Gender (male/female)	12/12	12/13	0.889
Age (years)	82.7 ± 7.3	82.3 ± 7.1	0.852
Active smoker (%)	0%	12%	0.248
Active drinker (%)	0%	20%	0.066
BMI (kg/m^2^)	22.4 ± 1.8	23.3 ± 2.3	0.915
Platelets (10^9^/L)	152.3 ± 108.3	179.6 ± 59.5	0.275
PT (s)	19.4 ± 8.1	15.3 ± 1.6	0.003
Total protein (g/L)	59.5 ± 8.3	74.0 ± 4.1	< 0.001
Albumin (g/L)	31.4 ± 5.1	44.5 ± 3.8	< 0.001
Globulin (g/L)	27.7 ± 6.0	29.6 ± 4.4	0.213
TB (μmol/L)	19.8 ± 19.3	15.3 ± 5.1	0.711
DB (μmol/L)	7.9 ± 9.9	4.2 ± 1.5	0.509
AST (U/L)	32.2 ± 17.5	17.6 ± 6.6	< 0.001
ALT (U/L)	22.0 ± 16.0	22.2 ± 5.4	0.114
GGT (U/L)	61.0 ± 51.6	33.6 ± 21.5	0.024
Child–Pugh			
A	13	/	NA
B	7	/	NA
C	4	/	NA

The continuous and categorical variables are listed as the mean ± SD and percentage (%), respectively. Statistical differences were determined using the Student’s *t*-test (parametric data) or the Wilcoxon Rank-Sum test (non-parametric data) and the *χ*^2^ test for continuous and categorical variables

*ALT* alanine aminotransferase, *AST* aspartate aminotransferase, *BMI* body mass index, *DB* direct bilirubin, *GGT* glutamyl transpeptidase, *PT* prothrombin time, *SLC* *S. japonicum* infection-induced liver cirrhosis,* TB* total bilirubin,* NA* not applicable

### Gut microbial alpha and beta diversity analysis between the two groups

**Fig. 1:**
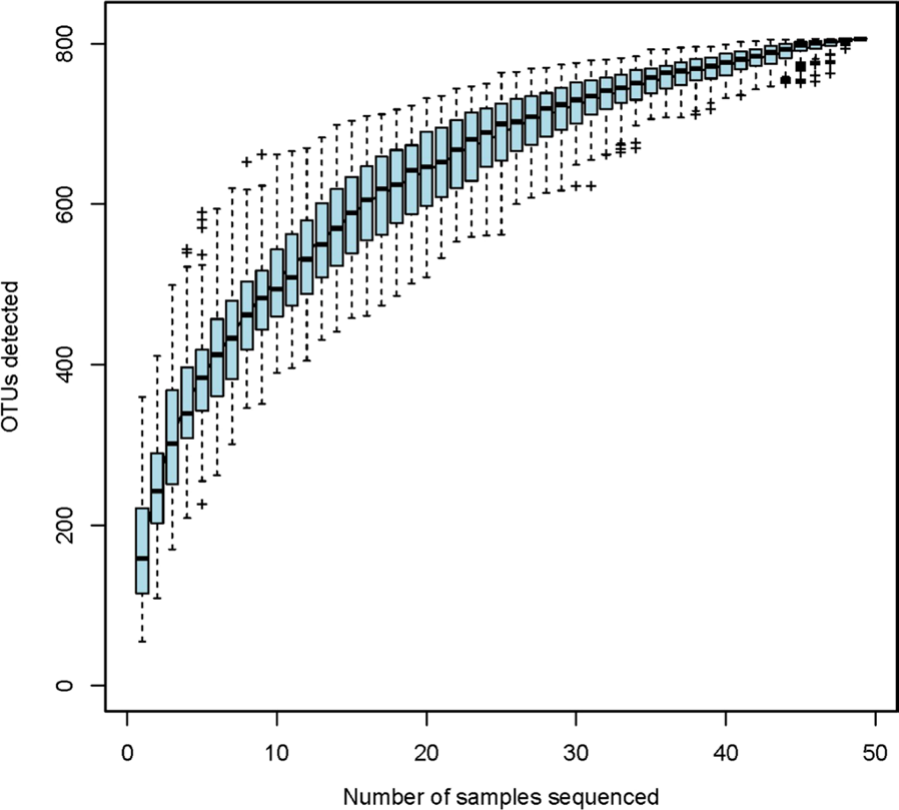
Accumulation curves attest to the sequencing depth adequacy. The accumulation curve is given by cumulative data of operational taxonomic units (OTUs) per sample. Each box and whisker plot represents the OTUs quantified for the respective number of samples sequenced (e.g., the first plot refers to one sample, while the second plot represents the cumulative data of two samples, etc.)

**Fig. 2:**
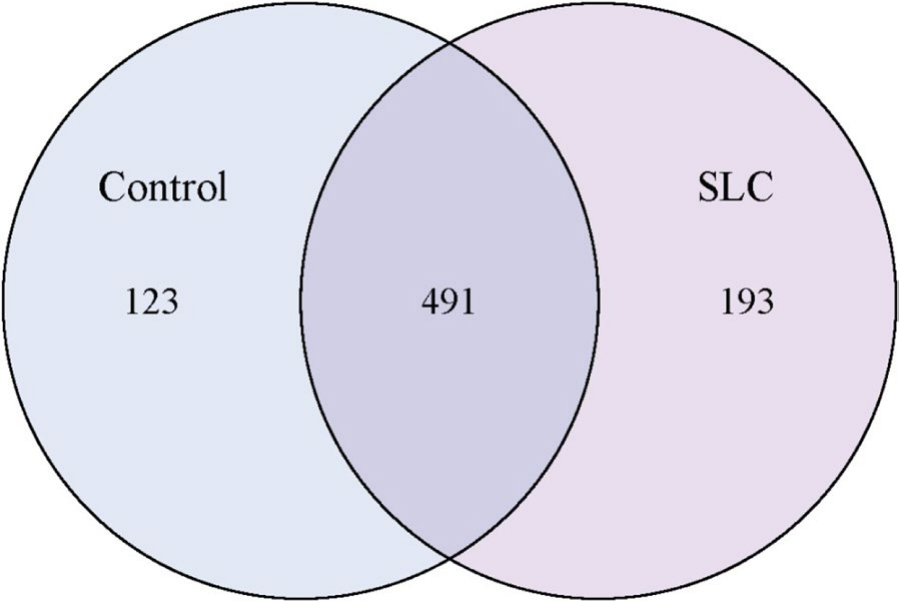
Representation of the operational taxonomic units (OTUs) detected per group of samples. The Venn diagram depicts the number of OTUs unique to healthy individuals (blue circle) and patients with *Schistosoma japonicum* infection-induced liver cirrhosis (SLC; purple circle), as well as those shared by the two groups (intersection of circles). SLC, *S. japonicum* infection-induced liver cirrhosis

The obtained accumulation curve reached a plateau, indicating that most of the gut microbial populations had been detected at the employed sequencing depth (Fig. 1). A total of 807 OTUs were obtained, of which 491 were equally detected in samples from cirrhosis individuals and healthy controls (Fig. 2). Notably, as represented in the Venn diagram, 123 and 193 OTUs were uniquely detected in healthy individuals and patients with *S. japonicum* infection-induced liver cirrhosis, respectively.

**Fig. 3:**
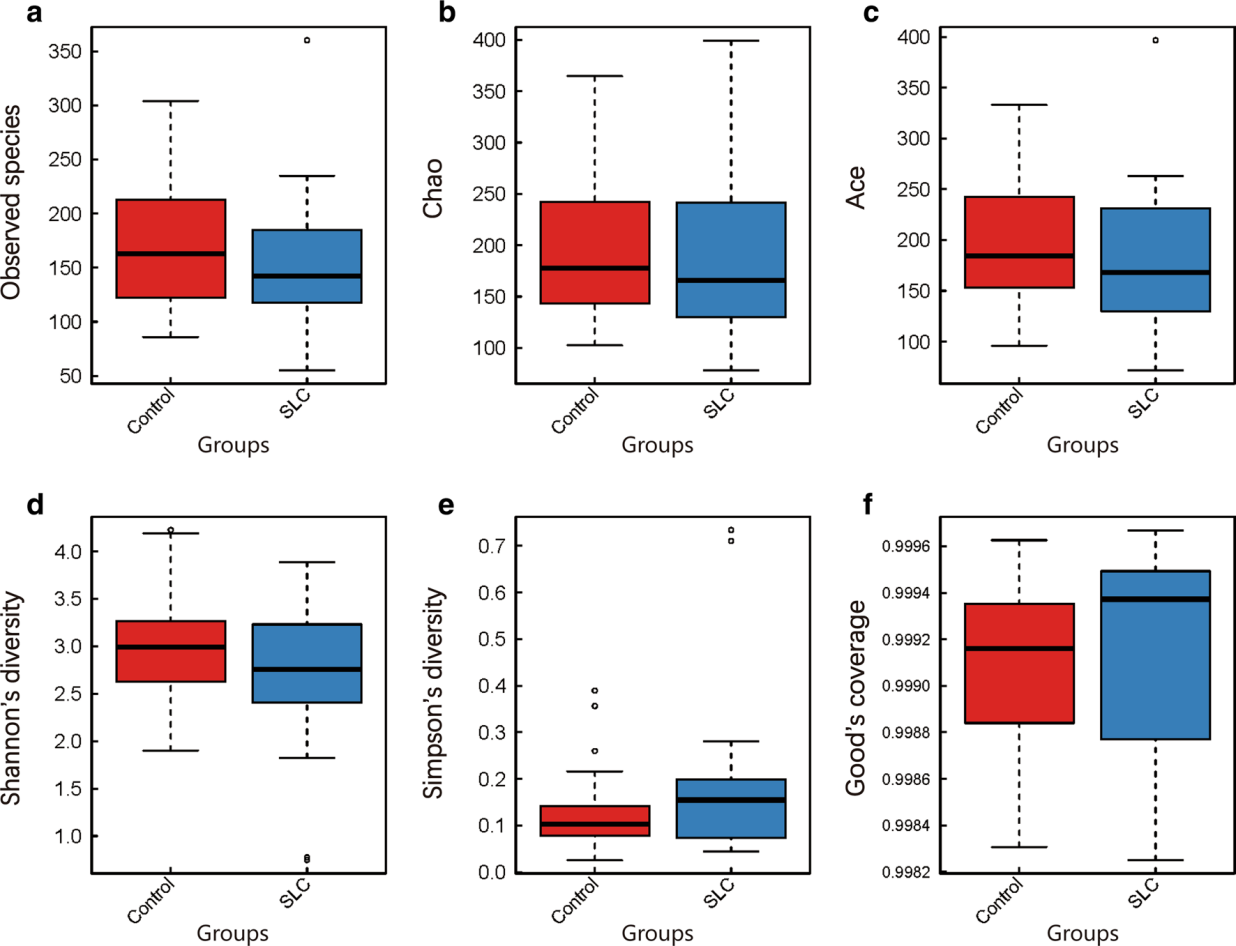
Alpha-diversity estimations are similar for control and cirrhosis groups. Intra-sample bacterial species diversity and richness were evaluated through the calculation of six different metrics. Results are presented as box and whisker plots per group (control and *Schistosoma japonicum* infection-induced liver cirrhosis—SLC—plots in red and blue, respectively) and per metric as follows: **a** Observed species; **b** Chao index; **c** ACE index; **d** Shannon index; **e** Simpson index; **f** Good-coverage index. No significant differences were detected between the groups. SLC, *S. japonicum* infection-induced liver cirrhosis

Concerning alpha diversity, we used six different metrics for analysis; specifically, the observed species, Chao, ACE, Shannon, Simpson, and Good’s coverage indexes were calculated. The resulting values were 173.4 ± 63.8, 197.7 ± 73.0, 196.3 ± 68.9, 2.96 ± 0.57, 0.13 ± 0.09, and 1.00 ± 0.00, respectively, in the control group and 154.0 ± 68.1, 178.6 ± 75.1, 179.9 ± 72.4, 2.68 ± 0.76, 0.19 ± 0.18, and 1.00 ± 0.00, respectively, in the cirrhosis group. Overall, we did not detect any significant changes when comparing healthy individuals to patients with *S. japonicum* infection-induced liver cirrhosis. However, a slight tendency toward a lower bacterial diversity was observed in the cirrhosis group (particularly for observed species, Chao, ACE, and Shannon indexes; Fig. 3). In addition, even considering the small number of cases showing disease severity in the cirrhosis group (13 Child–Pugh A, seven Child–Pugh B, and four Child–Pugh C cases), we further explored the variance in microbiota data by disease severity. The results had no statistical power likely due to the smaller sample size (particularly of the Child–Pugh C cohort); however, major trends could still be observed in Child–Pugh C patients (decreased Shannon index and increased Simpson index).

**Fig. 4:**
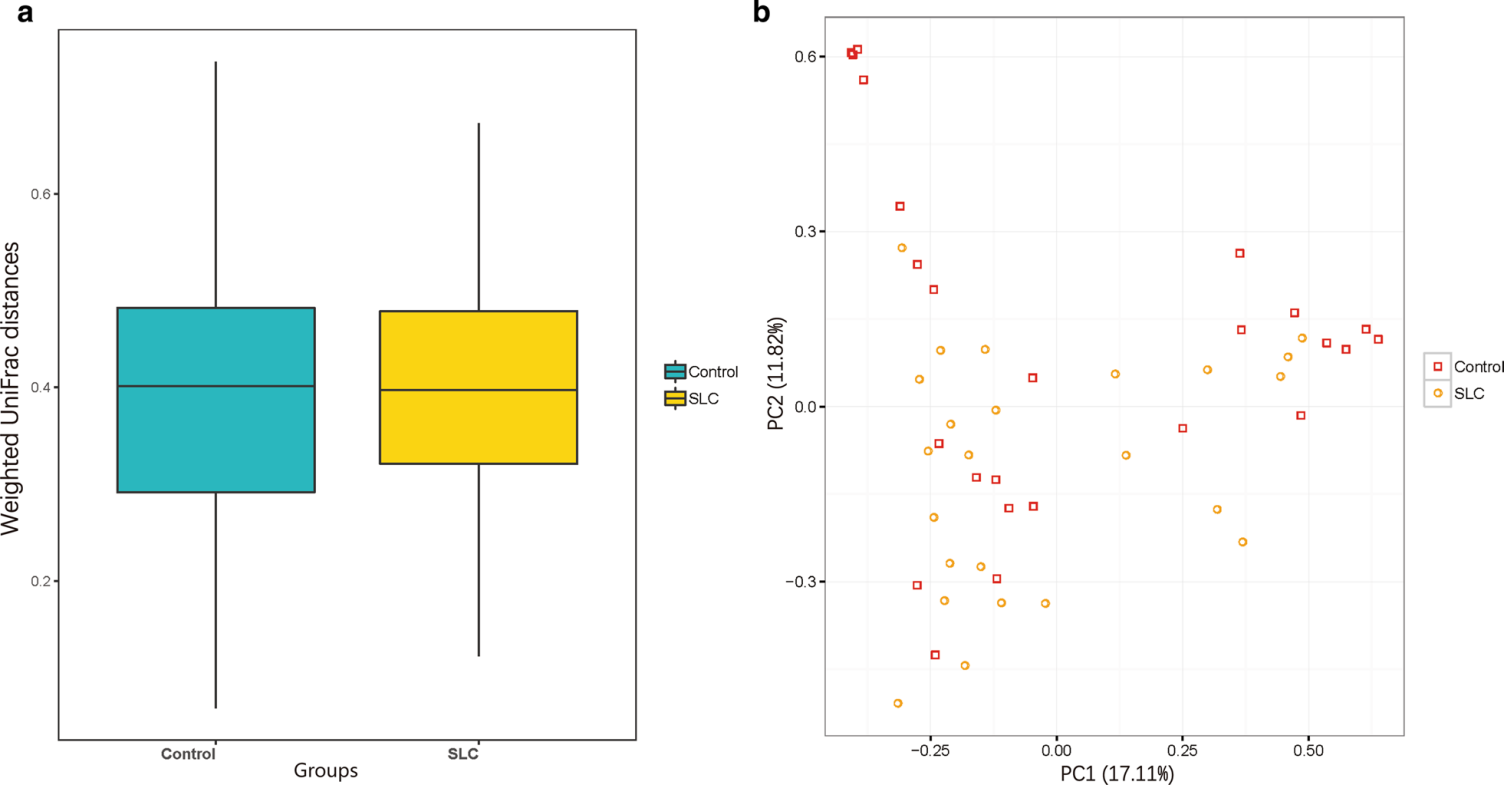
Gut microbiota diversity is not affected in patients with *Schistosoma japonicum* infection-induced liver cirrhosis (SLC). Beta diversity was calculated based on weighted UniFrac distances and is represented per group as box and whisker plots (**a**). A principal component analysis (PCA) was also performed and is presented (per sample, and group) (**b**). Yellow plot/symbols and blue or red plot/symbols refer to SLC and healthy control samples, respectively. In **a**, the *P*-value was determined using the Wilcoxon Rank-Sum test (*P* = 0.772). In **b**, we performed a PERMANOVA test (*P* = 0.09915). No statistical differences were detected between the two groups

We further analyzed beta diversity (based on weighted UniFrac distances), to evaluate differences between samples, and again, no major differences were observed between control and cirrhosis groups (0.40 ± 0.13 vs 0.40 ± 0.11; Fig. 4a). PCA data (Fig. 4b) confirmed this similarity. Samples from each group showed heterogeneous distributions that were not species-specific, indicating that the structure of gut bacterial communities is similar between healthy individuals and patients with *S. japonicum* infection-induced liver cirrhosis (Fig. 4b).

### Taxonomic composition of gut microbiota between the two groups

**Fig. 5:**
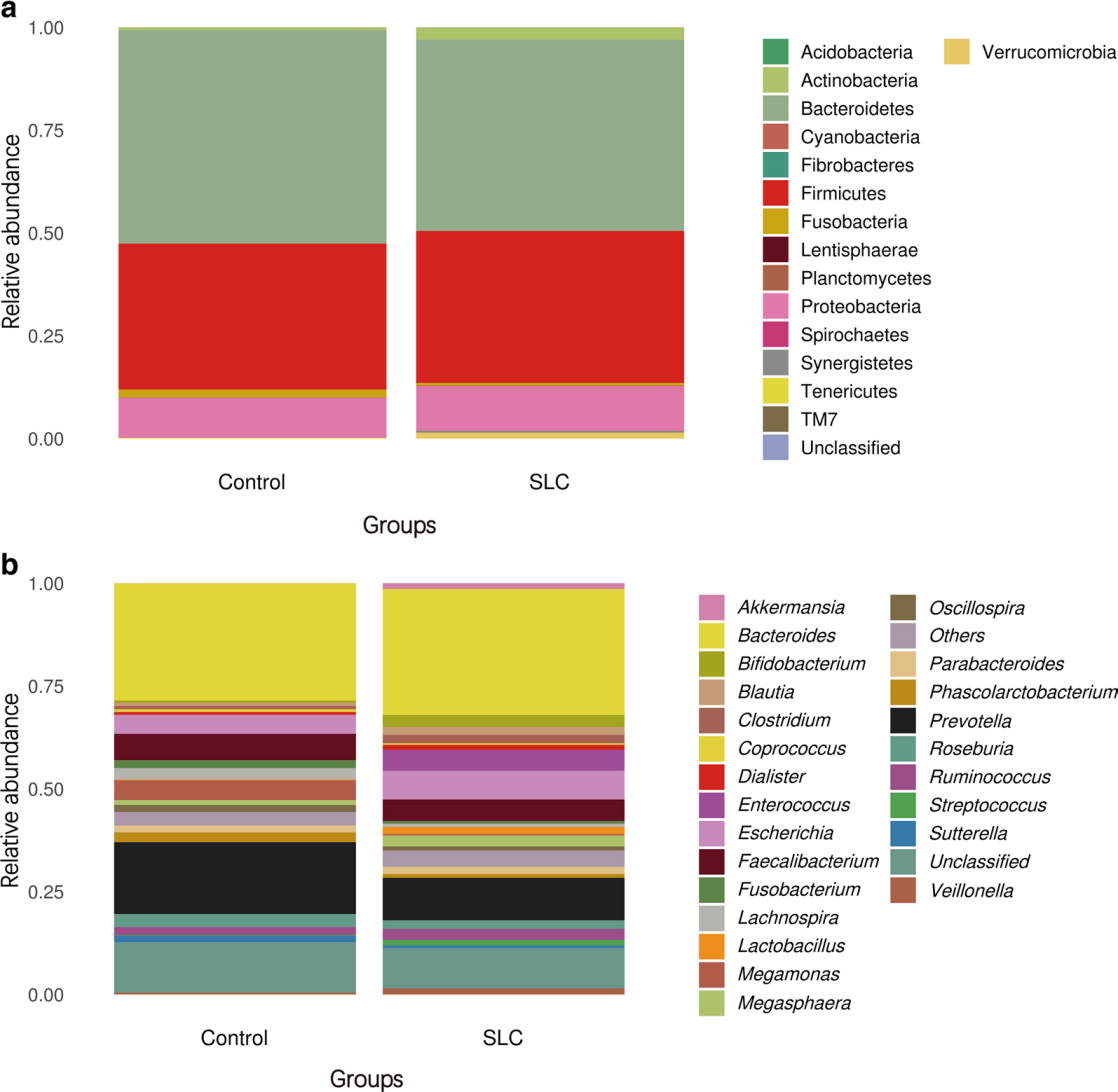
The gut bacterial composition is affected in *Schistosoma japonicum* infection-induced liver cirrhosis (SLC) patients. The relative abundances of bacterial phyla (**a**) and genera (**b**) detected in fecal samples were determined and are presented per group of samples. At the genus level, relative abundances of less than 0.5% were grouped and are presented as “others.” Phyla and genera are color-coded

We evaluated bacterial relative abundance at both phylum and genus levels (Fig. 5a and b, respectively). Analysis of the most abundant bacterial phyla in the fecal samples collected from the two groups revealed a similar pattern; specifically, Bacteroidetes, Firmicutes*,* and Proteobacteria were the three most prevalent phyla in both groups, accounting for 97.1% and 94.4% of all bacterial populations in healthy controls and cirrhosis patients, respectively (Fig. 5a). However, different patterns were observed at the genus level; specifically, *Bacteroides*, *Prevotella*, and *Faecalibacterium* were the three most prevalent genera in the guts of healthy individuals, whereas in cirrhosis patients, the third most prevalent genus was *Escherichia* (Fig. 5b).

**Fig. 6:**
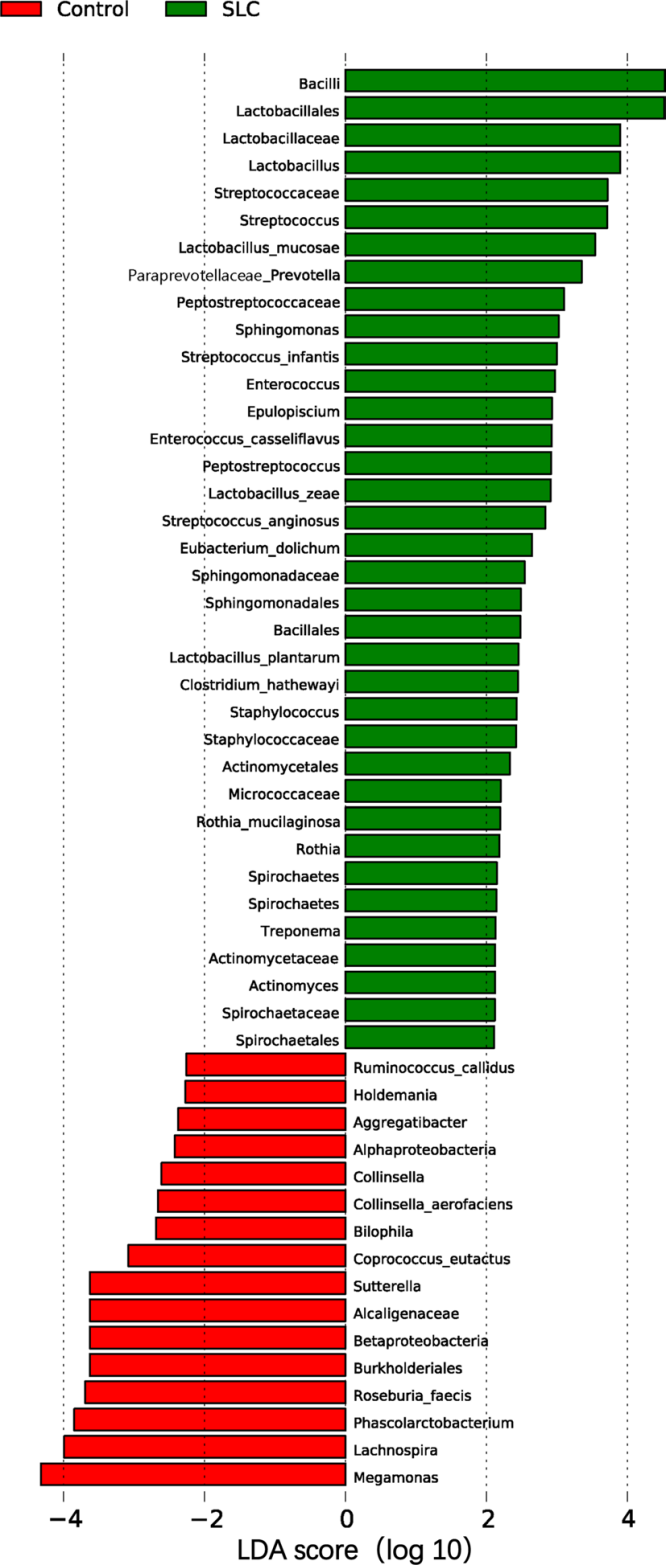
Taxonomic differences in the gut microbiotas of *Schistosoma japonicum* infection-induced liver cirrhosis (SLC) patients. The linear discriminant analysis (LDA) with the calculation of LDA effect size (LEfSe) was performed to identify the differentially abundant taxa between the two groups of samples, represented by red and green for healthy controls and SLC patients, respectively. The histogram of LDA scores shows the biomarkers with significant differences between groups; LDA absolute scores > 2 were considered statistically significant. The length of the column (LDA scores) represents the degree of influence of biomarkers. SLC, *S. japonicum* infection-induced liver cirrhosis; LDA, linear discriminant analysis

**Fig. 7:**
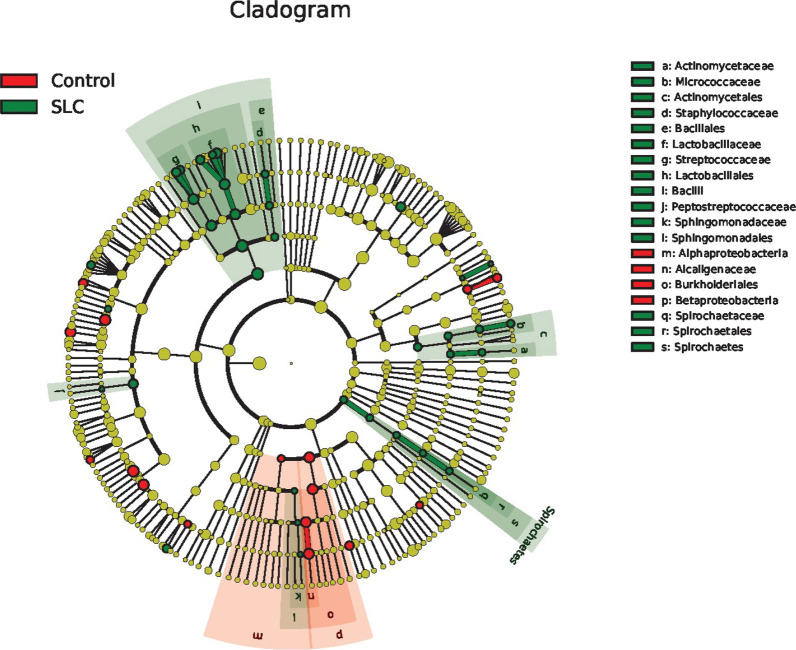
Gut microbiota community differences between healthy individuals and *Schistosoma japonicum* infection-induced liver cirrhosis (SLC) patients. The linear discriminant analysis (LDA) was translated into a cladogram that is presented. The red and green nodes represent specific microorganisms relevant to healthy controls and cirrhosis patients, respectively. Each node represents a biomarker; the biomarker names are included in the upper right corner. The yellow nodes represent the microorganisms that did not play an important role in the different groups. The diameter of each node is proportional to the taxon’s abundance. The circles that radiate from the inside to the outside represent the classification level from phylum to species (phylum–class–order–family–genus–species)

**Fig. 8:**
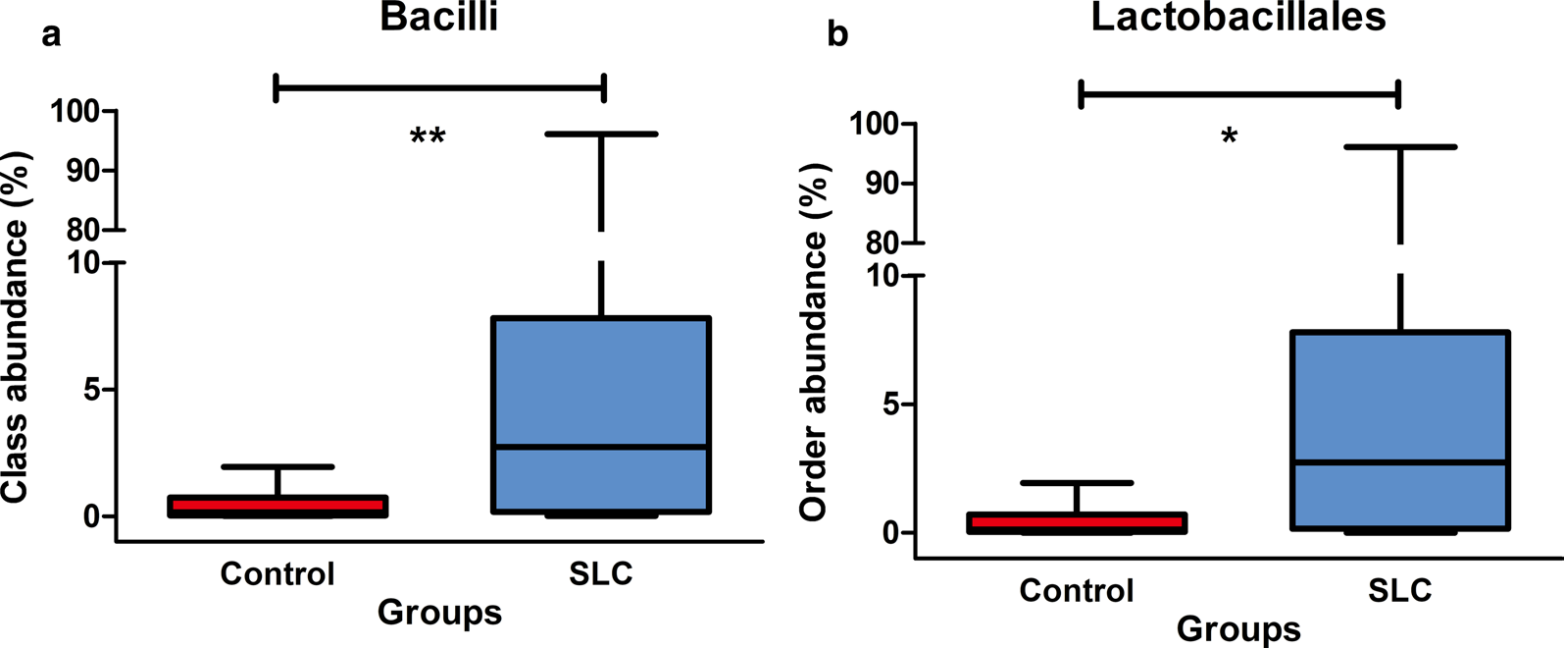
Bacilli class and Lactobacillales order are increased in *Schistosoma japonicum* infection-induced liver cirrhosis (SLC) patients. The relative abundances of relevant taxa in the gut of SLC patients at the class (**a**) and order (**b**) levels were calculated and are presented per group; red and blue columns refer to healthy individuals and SLC patients, respectively. Statistical significance was calculated and is represented: *FDR < 0.05, **FDR < 0.01. *FDR* false discovery rate

Additionally, to explore statistical relevance, we performed a LEfSe analysis, wherein *P* < 0.05 (Wilcoxon Rank-Sum test) and LDA scores > 2 were considered statistically significant (Figs. 6 and 7). Of note, we also considered FDR values in this analysis to minimize the number of false-positive results. Overall, we found no significant differences between the two groups at the phylum or genus levels, the most relevant categories in the context of taxonomic composition. Regarding the taxonomic composition of gut microbiota at other levels (class–order–family–species), only the Bacilli class and the Lactobacillales order were significantly increased in cirrhosis patients compared to those in healthy individuals (Fig. 8).

## Discussion

Liver cirrhosis is a common (end-stage) consequence of several liver diseases and a major cause of mortality worldwide [36]. Previous studies on gut-liver axis have demonstrated that the gut microbiota (and bacterial translocation) plays an important role in the pathogenesis of cirrhosis and its complications [37, 38]. However, few previous studies have explored the relationship between gut microbiota and *S. japonicum* infection-induced liver cirrhosis. Hence, this study explored the possible association between the gut microbiota and *S. japonicum* infection-induced liver cirrhosis in humans.

In this study, patients with liver cirrhosis showed increased levels of serum PT, AST, and GGT and decreased total protein, and albumin; of note, liver cirrhosis led to all of these changes. However, the globulin serum levels, defined previously as a good biomarker of inflammation and the immune status [39], did not change in the context of *S. japonicum* infection-induced liver cirrhosis. This result suggests that *S. japonicum* infection-induced liver cirrhosis might have a limited impact on systemic immune functions.

Surprisingly, we found that the microbiota of *S. japonicum* infection-induced liver cirrhosis patients was as rich and diverse as that of matched healthy individuals. Overall, these results are not consistent with findings reported in the context of in vivo studies. For example, in a mouse model of *S. japonicum* ova-induced granulomas, a significantly higher beta diversity was detected versus that in controls [24]. Similarly, an overall reduction in alpha and a significant increase in beta diversities were found in the guts of *S. mansoni*-infected mice compared to those in uninfected controls [22]. In our study, compared to healthy individuals, Child–Pugh C patients exhibited a decreased trend in the Shannon index and increased trend in the Simpson index. As such, an increase in the sample size is essential in future investigations to improve the statistical relevance and determine potential differences among groups.

Furthermore, the taxonomic composition at the phylum level was similar between cirrhosis patients and healthy individuals (prevalence of Bacteroidetes, Firmicutes*,* and Proteobacteria), aligning with in vivo data on *S. japonicum* infections [24]. Additionally, even at the genus level, no major changes were detected, indicating that in the context of our study, there is no gut dysbiosis in *S. japonicum* infection-induced liver cirrhosis patients. This result was unexpected and differed from that of other studies reporting gut dysbiosis in the context of human schistosomiasis (e.g., those caused by *S. mansoni* and *S. haematobium*) [21–25]. Additionally, in vivo studies have reported *Schistosoma* infection-related alterations in gut microbial composition. For example, *S. japonicum* infection in mice was found to be associated with significant changes in the gut prevalence of Firmicutes (relative decrease), Bacteroidetes, and Proteobacteria (relative increase) [24].

Moreover, no differences were observed between the two groups at the phylum or genus levels. However, when observed at other levels (class–order–family–species) differences were noted. Specifically, Bacilli (at the class level) and Lactobacillales (at the order level) were significantly increased in patients with *S. japonicum* infection-induced liver cirrhosis compared to levels in healthy individuals. Importantly, our results are consistent with those of a previous study, which might suggest that the increased prevalence of Lactobacillales in the gut microbiota is a hallmark of human liver cirrhosis [40]. Additionally, a different study showed that Lactobacillales and Bacilli were the key contributors to gut dysbiosis in primary liver cancer and proposed that they are potential diagnostic markers [41]. Taken together, these findings suggest a strong association between gut Bacilli and Lactobacillales and liver disease that warrants further exploration in different pathological contexts.

Overall, our data do not support the idea that *S. japonicum* infection-induced cirrhosis (in elderly individuals) is associated with significant alterations to gut microbiota profiles, opposing hypotheses proposed by others concerning schistosomiasis. A few reasons might justify our opposing results. For instance, studies have focused on different *Schistosoma* spp. with different organ tropisms; for example, *S. mansoni* and *S. japonicum* target the gut and liver, whereas *S. haematobium* targets the bladder and urogenital system [21–23, 25]. Importantly, the notion that *S. haematobium* infection is equally associated with human gut dysbiosis suggests that potential alterations in intestinal microbial communities in the context of schistosomiasis are likely a consequence of infection-induced systemic immune responses, not local events [21–23]. For example, mesenteric lymph node cells isolated from mice infected with *S. mansoni* (dysbiotic) respond differently than those isolated from controls, including higher interleukin (IL)-4, IL-10, and IL-17 secretion, together with lower interferon-γ production [23]. Appropriately, aging is accompanied by a deterioration of immune responses, recently named immunosenescence or immune-aging [42]. In our study, the enrolled patients were of advanced age (average of 82.7 ± 7.3 years), which might justify the absence of this phenotype; in other words, their potentially weaker immune responses to *S. japonicum* infection were not strong enough to impact their gut microbial communities. Of note, the serum globulin levels in liver cirrhosis patients did not change significantly, which could indirectly reflect the weaker effect of *S. japonicum* infection on systemic immunity in elderly individuals.

Nevertheless, our geographic target-region dictated the age range of our study population, which is a limitation of our study. Since 1995, due to the application of effective schistosomiasis control measures, there are almost no newly reported cases in Zhejiang [1]. Therefore, patients with *S. japonicum* infection-induced liver cirrhosis are not prevalent and tend to be of advanced ages [28]. Hence, we cannot exclude a possible age selection bias that reflects the reality of the population studied; specifically, among the cirrhosis patients, 17 and 7 individuals were 80 or 90 years of age or older, respectively, and none of the others was less than 60 years of age. We can, however, also hypothesize that our population has a survival bias. Living patients could be those with better outcomes due to stable gut microbiota profiles, whereas prior fatalities might have been a consequence of gut microbiota disorders. This is, however, only speculative. Therefore, further studies focusing on much larger and heterogeneous populations (different geographies, with a wider age range) are needed to fully elucidate whether *S. japonicum* infection-induced liver cirrhosis is accompanied by gut microbiota alterations. In addition, the effective schistosomiasis control in Zhejiang and advanced age of participants might affect our conclusion because it was almost impossible to include a cohort of age-matched *S. japonicum* acutely infected patients without liver cirrhosis.

An additional factor that should be considered in future studies is the timing of infection, particularly if the study is designed to identify biomarkers for the diagnosis and/or prognosis of cirrhosis. Indeed, the infection status might also determine the results. Human studies have primarily targeted adolescents [21] or children [25], and consequently, the early phase of infection. Additionally, the most commonly used murine model of schistosomiasis in microbiome studies can be used to evaluate acute infection, specifically, less than eight weeks post-challenge according to the model characterization [43–45]. The in vivo published data refer to stool samples collected at 28 days [22], 42 days [24], and 8 weeks [23] after infection. Hence, we cannot exclude the possibility that our conflicting findings are simply a consequence of our study context. Cirrhosis is the end-stage of chronic schistosomiasis that occurs significantly later than the early phase of infection. Both systemic immune responses and *Schistosoma* infection were found to be associated with gut microbiota alterations [21]. However, in *S. japonicum* infection-induced liver cirrhosis, both are expected to decrease in magnitude, which might partially explain our findings.

## Conclusions

Overall, the results show that the gut microbial taxonomic profiles and diversity in elderly *S. japonicum* infection-induced liver cirrhosis patients appear to be similar to those from age-matched healthy individuals. Therefore, in our study, patients with *S. japonicum* infection-induced liver cirrhosis present a “healthy” gut structure, which might partially explain the better prognosis of cirrhosis secondary to this infection, as compared to that with cirrhosis of different etiologies in China. Future studies with a much larger sample size and considering a wider age range are, however, needed to further explore the relationship between *S. japonicum* infection-induced liver cirrhosis and gut microbiota, and potentially disclose the possible mechanisms by which gut microbiota alterations influence cirrhosis and/or vice versa. Importantly, these studies should also evaluate immunity and inflammation to finally determine if the gut-liver axis is implicated in *S. japonicum* infection-induced liver cirrhosis development and progression. Such studies should be further complemented by in vivo works with the transplantation of human gut microbiota samples to ultimately establish (or exclude) a causal relationship between dysbiosis and cirrhosis secondary to schistosomiasis.

## Data Availability

The data supporting the conclusions of this article are included in the manuscript. The datasets analyzed during the present study are available from the corresponding author upon reasonable request.

## Abbreviations

OTUs: Operational taxonomic units
IQR: Interquartile range
TB: Total bilirubin
PT: Prothrombin time
PCR: Polymerase chain reaction
CT: Computed tomography
PCA: Principal component analysis
LDA: Linear discriminant analysis
LEfSe: LDA effect size
*SD*: Standard deviation
FDR: False discovery rate
AST: Aspartate aminotransferase
GGT: Glutamyl transpeptidase
SLC: *S. japonicum* Infection-induced liver cirrhosis
IL: Interleukin

## References

[1] Cao ZG, Zhao YE, Lee Willingham A, Wang TP (2016). Towards the elimination of schistosomiasis japonica through control of the disease in domestic animals in the People's Republic of China: a tale of over 60 years. Adv Parasitol.

[2] World Health Organization. World Health Organisation Schistosomiasis (Bilharzia). World Health Organization. 2021. https://www.who.int/health-topics/schistosomiasis#tab=tab_1. Accessed 24 Jan 2021.

[3] Steinmann P, Keiser J, Bos R, Tanner M, Utzinger J (2006). Schistosomiasis and water resources development: systematic review, meta-analysis, and estimates of people at risk. Lancet Infect Dis.

[4] Murray CJ, Vos T, Lozano R, Naghavi M, Flaxman AD, Michaud C (2012). Disability-adjusted life years (DALYs) for 291 diseases and injuries in 21 regions, 1990–2010: a systematic analysis for the Global Burden of Disease Study 2010. Lancet.

[5] Chuah C, Gobert GN, Latif B, Heo CC, Leow CY (2019). Schistosomiasis in Malaysia: a review. Acta Trop.

[6] Zhang SQ, Sun CS, Wang M, Lin DD, Zhou XN, Wang TP (2016). Epidemiological features and effectiveness of schistosomiasis control programme in lake and marshland region in the People's Republic of China. Adv Parasitol.

[7] Li ZJ, Ge J, Dai JR, Wen LY, Lin DD, Madsen H (2016). Biology and control of snail intermediate host of *Schistosoma japonicum* in the People's Republic of China. Adv Parasitol.

[8] Carson JP, Ramm GA, Robinson MW, McManus DP, Gobert GN (2018). Schistosome-induced fibrotic disease: the role of hepatic stellate cells. Trends Parasitol.

[9] Anthony B, Allen JT, Li YS, McManus DP (2010). Hepatic stellate cells and parasite-induced liver fibrosis. Parasit Vectors.

[10] Seki E, Brenner DA (2015). Recent advancement of molecular mechanisms of liver fibrosis. J Hepatobiliary Pancreat Sci.

[11] Kamdem SD, Moyou-Somo R, Brombacher F, Nono JK (2018). Host regulators of liver fibrosis during human schistosomiasis. Front Immunol.

[12] Wei X, Jiang S, Chen Y, Zhao X, Li H, Lin W (2016). Cirrhosis related functionality characteristic of the fecal microbiota as revealed by a metaproteomic approach. BMC Gastroenterol.

[13] Schwimmer JB, Johnson JS, Angeles JE, Behling C, Belt PH, Borecki I (2019). Microbiome signatures associated with steatohepatitis and moderate to severe fibrosis in children with nonalcoholic fatty liver disease. Gastroenterology.

[14] Bajaj JS, Thacker LR, Fagan A, White MB, Gavis EA, Hylemon PB (2018). Gut microbial RNA and DNA analysis predicts hospitalizations in cirrhosis. JCI Insight.

[15] Heidrich B, Vital M, Plumeier I, Doscher N, Kahl S, Kirschner J (2018). Intestinal microbiota in patients with chronic hepatitis C with and without cirrhosis compared with healthy controls. Liver Int.

[16] Loomba R, Seguritan V, Li W, Long T, Klitgord N, Bhatt A (2017). Gut microbiome-based metagenomic signature for non-invasive detection of advanced fibrosis in human nonalcoholic fatty liver disease. Cell Metab.

[17] Bajaj JS, Heuman DM, Hylemon PB, Sanyal AJ, White MB, Monteith P (2014). Altered profile of human gut microbiome is associated with cirrhosis and its complications. J Hepatol.

[18] Bajaj JS, Betrapally NS, Hylemon PB, Heuman DM, Daita K, White MB (2015). Salivary microbiota reflects changes in gut microbiota in cirrhosis with hepatic encephalopathy. Hepatology.

[19] Ling Z, Liu X, Cheng Y, Jiang X, Jiang H, Wang Y (2015). Decreased diversity of the oral microbiota of patients with hepatitis b virus-induced chronic liver disease: a pilot project. Sci Rep.

[20] Oikonomou T, Papatheodoridis GV, Samarkos M, Goulis I, Cholongitas E (2018). Clinical impact of microbiome in patients with decompensated cirrhosis. World J Gastroenterol.

[21] Ajibola O, Rowan AD, Ogedengbe CO, Mshelia MB, Cabral DJ, Eze AA (2019). Urogenital schistosomiasis is associated with signatures of microbiome dysbiosis in Nigerian adolescents. Sci Rep.

[22] Jenkins TP, Peachey LE, Ajami NJ, MacDonald AS, Hsieh MH, Brindley PJ (2018). *Schistosoma mansoni* infection is associated with quantitative and qualitative modifications of the mammalian intestinal microbiota. Sci Rep.

[23] Floudas A, Aviello G, Schwartz C, Jeffery IB, O'Toole PW, Fallon PG (2019). *Schistosoma mansoni* worm infection regulates the intestinal microbiota and susceptibility to colitis. Infect Immun.

[24] Zhao Y, Yang S, Li B, Li W, Wang J, Chen Z (2019). Alterations of the mice gut microbiome via *Schistosoma japonicum* ova-induced granuloma. Front Microbiol.

[25] Kay GL, Millard A, Sergeant MJ, Midzi N, Gwisai R, Mduluza T (2015). Differences in the faecal microbiome in *Schistosoma haematobium* infected children vs. uninfected children. PLoS Negl Trop Dis.

[26] Schneeberger PHH, Coulibaly JT, Panic G, Daubenberger C, Gueuning M, Frey JE (2018). Investigations on the interplays between *Schistosoma mansoni*, praziquantel and the gut microbiome. Parasit Vectors.

[27] Wu W, Feng A, Huang Y (2015). Research and control of advanced schistosomiasis japonica in China. Parasitol Res.

[28] Song LG, Wu XY, Sacko M, Wu ZD (2016). History of schistosomiasis epidemiology, current status, and challenges in China: on the road to schistosomiasis elimination. Parasitol Res.

[29] Cholongitas E, Papatheodoridis GV, Vangeli M, Terreni N, Patch D, Burroughs AK (2005). Systematic review: the model for end-stage liver disease–should it replace Child-Pugh's classification for assessing prognosis in cirrhosis?. Aliment Pharmacol Ther.

[30] Magoc T, Salzberg SL (2011). FLASH: fast length adjustment of short reads to improve genome assemblies. Bioinformatics.

[31] Edgar RC (2013). UPARSE: highly accurate OTU sequences from microbial amplicon reads. Nat Methods.

[32] Edgar RC, Haas BJ, Clemente JC, Quince C, Knight R (2011). UCHIME improves sensitivity and speed of chimera detection. Bioinformatics.

[33] Caporaso JG, Kuczynski J, Stombaugh J, Bittinger K, Bushman FD, Costello EK (2010). QIIME allows analysis of high-throughput community sequencing data. Nat Methods.

[34] Edgar RC (2010). Search and clustering orders of magnitude faster than BLAST. Bioinformatics.

[35] Schloss PD, Westcott SL, Ryabin T, Hall JR, Hartmann M, Hollister EB (2009). Introducing mothur: open-source, platform-independent, community-supported software for describing and comparing microbial communities. Appl Environ Microbiol.

[36] Lozano R, Naghavi M, Foreman K, Lim S, Shibuya K, Aboyans V (2012). Global and regional mortality from 235 causes of death for 20 age groups in 1990 and 2010: a systematic analysis for the Global Burden of Disease Study 2010. Lancet.

[37] Arab JP, Martin-Mateos RM, Shah VH (2018). Gut-liver axis, cirrhosis and portal hypertension: the chicken and the egg. Hepatol Int.

[38] Acharya C, Bajaj JS (2017). Gut Microbiota and complications of liver disease. Gastroenterol Clin North Am.

[39] Zhou Q, Cao H, Xu Z, Lan R, Chen X, Wang D (2017). Baseline serum globulin as a predictor of the recurrence of lone atrial fibrillation after radiofrequency catheter ablation. Anatol J Cardiol.

[40] Bajaj JS, Ahluwalia V, Steinberg JL, Hobgood S, Boling PA, Godschalk M (2016). Elderly patients have an altered gut-brain axis regardless of the presence of cirrhosis. Sci Rep.

[41] Zhang L, Wu YN, Chen T, Ren CH, Li X, Liu GX (2019). Relationship between intestinal microbial dysbiosis and primary liver cancer. Hepatobiliary Pancreat Dis Int.

[42] Walford RL (1964). The immunologic theory of aging. Gerontologist.

[43] Ferrari TC, Moreira PR (2011). Neuroschistosomiasis: clinical symptoms and pathogenesis. Lancet Neurol.

[44] Seki T, Kumagai T, Kwansa-Bentum B, Furushima-Shimogawara R, Anyan WK, Miyazawa Y (2012). Interleukin-4 (IL-4) and IL-13 suppress excessive neutrophil infiltration and hepatocyte damage during acute murine schistosomiasis japonica. Infect Immun.

[45] Chen L, Chen Q, Hou W, He L (2017). High-throughput dynamic analysis of differentially expressed genes in splenic dendritic cells from mice infected with *Schistosoma japonicum*. Immunol Lett.

